# Supplementary material for: Exploring the association of genetic factors with participation in the Avon Longitudinal Study of Parents and Children
Source: Int J Epidemiol. 2018 May 23;47(4):1207–16. doi: 10.1093/ije/dyy060 (PMC6124613; doi:10.1093/ije/dyy060)
Supplement: Supplementary Data [file dyy060_supplementary_methods.docx]

**Supplementary methods**

**GWAS data generation and quality control**

ALSPAC children were genotyped using the Illumina HumanHap550 quad chip genotyping platforms. The resulting raw genome-wide data were subjected to standard quality control methods. Individuals were excluded on the basis of gender mismatches; minimal or excessive heterozygosity; disproportionate levels of individual missingness (>3%) and insufficient sample replication (IBD < 0.8). Population stratification was assessed by multidimensional scaling analysis and compared with Hapmap II (release 22) European descent (CEU), Han Chinese, Japanese and Yoruba reference populations; all individuals with non-European ancestry were removed. SNPs with a minor allele frequency of < 1%, a call rate of < 95% or evidence for violations of Hardy-Weinberg equilibrium (P < 5E-7) were removed. Cryptic relatedness was measured as proportion of identity by descent (IBD > 0.1). Related subjects that passed all other quality control thresholds were retained during subsequent phasing and imputation. 9,115 subjects and 500,527 SNPs passed these quality control filters.

ALSPAC mothers were genotyped using the Illumina human660W-quad array at Centre National de Génotypage (CNG) and genotypes were called with Illumina GenomeStudio. PLINK (v1.07) was used to carry out quality control measures on an initial set of 10,015 subjects and 557,124 directly genotyped SNPs. SNPs were removed if they displayed more than 5% missingness or a Hardy-Weinberg equilibrium P value of less than 1.0e-06. Additionally, SNPs with a minor allele frequency of less than 1% were removed. Samples were excluded if they displayed more than 5% missingness, had indeterminate X chromosome heterozygosity or extreme autosomal heterozygosity. Samples showing evidence of population stratification were identified by multidimensional scaling of genome-wide identity by state pairwise distances using the four HapMap populations as a reference, and then excluded. Cryptic relatedness was assessed using a IBD estimate of more than 0.125 which is expected to correspond to roughly 12.5% alleles shared IBD or a relatedness at the first cousin level. Related subjects that passed all other quality control thresholds were retained during subsequent phasing and imputation. 9,048 subjects and 526,688 SNPs passed these quality control filters.

After combining genotype data in the mothers and the children, SNPs with genotype missingness above 1% were removed due to poor quality (11,396 SNPs removed) and a further 321 subjects were removed due to potential ID mismatches. This resulted in a dataset of 17,842 subjects. Imputation of the target data was performed using Impute V2.2.2 against the 1000 genomes reference panel (Phase 1, Version 3) (all polymorphic SNPs excluding singletons), using all 2186 reference haplotypes (including non-Europeans).

This gave 8,237 eligible children and 8,196 eligible mothers with available genotype data after exclusion of related subjects using cryptic relatedness measures described previously.

**PRSice**

We used the PRSice software (<http://prsice.info/>) to generate polygenic scores for each trait within the ALSPAC genome-wide data. PRSice performs linkage disequilibrium clumping based on SNPs available in both GWAS and target datasets. We excluded SNPs with an R^2^ of >0.1, which were within 250Kb of each other. SNPs located in the extended MHC region (chromosome 6 (26-33Mb)) were excluded. We used the following p-value thresholds to define polygenic scores: 0.0005, 0.005, 0.05, 0.1, 0.5. Analyses were performed separately in mothers and children using bestguess genotypes generated from the imputed data, filtered on a minor allele frequency >0.01 and imputation quality of >0.8.

|  | **Mother questionnaires** | **Mother clinics** | **Child based questionnaires** | **Child completed questionnaires** | **Child clinics** |
| --- | --- | --- | --- | --- | --- |
| 1 | 8-42 weeks gestation | 17-18 years | 4 weeks | 65 months | 7 years |
| 2 | 12 weeks gestation |  | 6 months | 69 months | 8 years |
| 3 | 18 weeks gestation |  | 15 months | 73 months | 9 years |
| 4 | 32 weeks gestation |  | 18 months | 77 months | 10 + years |
| 5 | 8 weeks |  | 24 months | 81 months | 11 + years |
| 6 | 8 months |  | 30 months | 85 months | 13 years |
| 7 | 21 months |  | 38 months | 91 months | 14 years |
| 8 | 33 months |  | 42 months | 97 months | 15 years |
| 9 | 47 months |  | 54 months | 103 months | 18 years |
| 10 | 61 months |  | 57 months | 110 months |  |
| 11 | 85 months |  | 65 months | 115 months |  |
| 12 | 73 months |  | 69 months | 122 months |  |
| 13 | 97 months |  | 77 months | 128 months |  |
| 14 | 110 months |  | 81 months | 134 months |  |
| 15 | 122 months |  | 91 months | 140 months |  |
| 16 | 134 months |  | 103 months | 145 months |  |
| 17 | 145 months |  | 103 months | 157 months |  |
| 18 | 18 years + |  | 115 months | 157 months |  |
| 19 | 19 years + |  | 128 months | 166 months |  |
| 20 |  |  | 140 months | 167 months |  |
| 21 |  |  | 157 months | 169 months |  |
| 22 |  |  | 166 months | 198 months |  |
| 23 |  |  | 198 months | 18 years |  |
| 24 |  |  | 19 years + | 20 years |  |

**Table S1. Timepoints of questionnaires and clinics included in participation phenotype**

**Table S2. Description of polygenic risk scores**

| **Trait** | **Reference** | **Data available at:** |
| --- | --- | --- |
| Body mass index | Locke et al. (*1*) | http://portals.broadinstitute.org/collaboration/giant/index.php/GIANT_consortium_data_files |
| Height | Wood et al. (*2*) | http://portals.broadinstitute.org/collaboration/giant/index.php/GIANT_consortium_data_files |
| Smoking initiation | Furberg et al. (*3*) | https://www.med.unc.edu/pgc |
| Education | Okbay et al. (*4*) | https://www.thessgac.org/data |
| Sleep | Jones et al. (*5*) | http://www.t2diabetesgenes.org/data/ |
| Morningness | Jones et al. (*5*) | http://www.t2diabetesgenes.org/data/ |
| Age at Menarche | Perry et al. (*6*) | http://www.reprogen.org/data_download.html |
| Depression | Okbay et al. (*7*) | https://www.thessgac.org/data |
| Schizophrenia | Schizophrenia working group of Psychiatric Genomics Consortium (*8*) | https://www.med.unc.edu/pgc |
| ADHD | Psychiatric Genomics Consortium (PGC) and the Lundbeck Foundation Initiative for Integrative Psychiatric Research (iPSYCH) (*9*) | https://www.med.unc.edu/pgc |
| Bipolar | Cross-Disorder Group of the Psychiatric Genomics Consortium (*10*) |  |
| Autism |  |  |
| Agreeableness | De Moor et al. (*11*) | http://www.tweelingenregister.org/GPC/ |
| Conscientiousness |  |  |
| Extraversion |  |  |
| Openness |  |  |
| Neuroticism |  |  |
| Alzheimer’s Disease | Lambert et al. (*12*) | http://web.pasteur-lille.fr/en/recherche/u744/igap/igap_download.php |

**Table S3 Genomewide scores for traits generate in PRSice**

| **Trait** | **Number of independent genomewide SNPs in original GWAS paper** | **Number in score generate using PRSice** |
| --- | --- | --- |
| Years of Education | 74 | 64 |
| BMI | 77 (in Europeans) | 72 |
| Smoking initiation | 1 | 1 |
| Morningness | 11 (in discovery sample which were replicated) | 7 |
| Sleep | 3 | 2 |
| Age at menarche | 123 | 108 |
| Alzheimer’s | 19 (in discovery sample which were replicated) | 18 |
| ADHD | 12 (in full ADHD GWAS) | 8 |
| Depression | 2 | 2 |
| Schizophrenia | 128 | 95 |
| Height | 697 | 535 |
| Autism | 1 | 1 |
| Bipolar | 4 | 4 |
| Conscientiousness | 1 | 1 |
| Openness | 2 | 1 |

**Table S4 Dichomisation point for sensitivity analyses using continuous variables**

| **Individual** | **Metric** | **Model** | **Dichotomisation point** |
| --- | --- | --- | --- |

| Mother | Mother questionnaire | Logistic | 12 |
| --- | --- | --- | --- |
|  | Total questionnaire |  | 30 |
|  | Total participation |  | 30 |
|  | Most recent clinic |  | NA |
|  | Most recent questionnaire |  | NA |
| Child | Child questionnaire | Logistic | 5 |
|  | Total questionnaire |  | 30 |
|  | Total participation |  | 30 |
|  | Child clinic |  | 4 |
|  | Most recent clinic |  | NA |
|  | Most recent questionnaire |  | NA |

|  | **Mother** | | **Child** | |
| --- | --- | --- | --- | --- |
|  | **Analysis sample**  **(N=7,486)** | **Full sample**  **(N=13,793)** | **Analysis sample**  **(N=7,508)** | **Full sample**  **(N=13,953)** |
|  | **Mean (SD)** | **Mean (SD)** | **Mean (SD)** | **Mean (SD)** |
| **Total Participation** | 50.6 (23.8) | 44.0 (25.5) | 53.5 (22.0) | 44.0 (25.4) |
| **Total Questionnaire** | 45.0 (20.7) | 39.5 (22.2) | 47.4 (19.2) | 39.4 (22.2) |
| **Mother Questionnaire** | 14.0 (5.2) | 12.7 (5.7) | - | - |
| **Child Questionnaire** | - | - | 14.8 (8.0) | 11.7 (8.8) |
| **Child Clinic** | - | - | 5.7 (3.4) | 4.2 (3.7) |
|  | **N (%)** | **N (%)** | **N (%)** | **N (%)** |
| **Mother attended most recent clinic** | 3,215 (43.0) | 4,634 (33.6) | - | - |
| **Mother completed most recent questionnaire** | 3,052 (40.8) | 4,468 (32.4) | - | - |
| **Child attended most recent clinic** | - |  | 3,538 (47.1) | 4,878 (35.0) |
| **Child completed most recent questionnaire** | - |  | 2,957 (39.4) | 4,147 (29.7) |

**Table S5. Comparison of analysis sample with full sample.**

|  | **Child** | | | | **Mother** | | | |
| --- | --- | --- | --- | --- | --- | --- | --- | --- |
|  | Total | Total questionnaire | Child questionnaire | Child clinic |  | Total | Total questionnaire | Mother questionnaire |
| Total | 1 |  |  |  | Total | 1 |  |  |
| Total questionnaire | 0.9867 | 1 |  |  | Total questionnaire | 0.9877 | 1 |  |
| Child questionnaire | 0.9505 | 0.9611 | 1 |  | Mother questionnaire | 0.9379 | 0.948 | 1 |
| Child clinic | 0.8093 | 0.7202 | 0.7193 | 1 |  |  |  |  |

**Table S6. Correlations between continuous participation phenotypes**

Correlation coefficients are Spearman’s rank correlations

**Table S7. Pearson’s correlation coefficients between genetic risk scores generated at p<0.5 in the ALSPAC mothers (N=7,468)**

**Table S8. Pearson’s correlation coefficients between genetic risk scores generated at p<0.0005 in the ALSPAC mothers (N=7,468)**

**Table S9. Pearson’s correlation coefficients between genetic risk scores generated at p<0.5 in the ALSPAC children (N=7,508)**

**Correlations with age at menarche in females (N=3,639)**

**Table S10. Pearson’s correlation coefficients between genetic risk scores generated at p<0.0005 in the ALSPAC children (N=7,508)**

**Correlations with age at menarche in females (N=3,639)**

**Table S11. Genomewide significant associations with total participation in mothers**

| **SNP** | **CHR** | **BP** | **A1** | **A2** | **Beta** | **SE** | **P** | **A1_freq** | **info** |
| --- | --- | --- | --- | --- | --- | --- | --- | --- | --- |
| rs10626545 | 7 | 51999752 | TCA | T | 10.97 | 1.81 | 1.50E-09 | 0.987 | 0.94 |
| rs406001 | 7 | 52006415 | C | T | 10.86 | 1.80 | 1.67E-09 | 0.988 | 1.00 |
| rs10234957 | 7 | 52011171 | A | G | 10.83 | 1.81 | 2.12E-09 | 0.988 | 1.00 |
| rs10265236 | 7 | 52011178 | T | C | 10.83 | 1.81 | 2.12E-09 | 0.988 | 1.00 |
| rs392252 | 7 | 52039815 | G | A | 10.84 | 1.81 | 2.15E-09 | 0.988 | 1.00 |
| rs413648 | 7 | 52013580 | G | T | 10.81 | 1.81 | 2.23E-09 | 0.988 | 1.00 |
| rs403531 | 7 | 52007845 | G | A | 10.81 | 1.81 | 2.23E-09 | 0.988 | 1.00 |
| rs616199 | 7 | 52014884 | C | A | 10.81 | 1.81 | 2.23E-09 | 0.988 | 1.00 |
| rs428360 | 7 | 52015414 | A | T | 10.81 | 1.81 | 2.23E-09 | 0.988 | 1.00 |
| rs410473 | 7 | 52015969 | A | G | 10.81 | 1.81 | 2.24E-09 | 0.988 | 1.00 |
| rs382903 | 7 | 52033199 | A | G | 10.81 | 1.81 | 2.24E-09 | 0.988 | 1.00 |
| rs377851 | 7 | 52031652 | T | A | 10.81 | 1.81 | 2.24E-09 | 0.988 | 1.00 |
| rs364752 | 7 | 52021977 | T | C | 10.81 | 1.81 | 2.25E-09 | 0.988 | 1.00 |
| rs417388 | 7 | 52029191 | T | C | 10.81 | 1.81 | 2.25E-09 | 0.988 | 1.00 |
| rs454526 | 7 | 52030332 | C | A | 10.81 | 1.81 | 2.25E-09 | 0.988 | 1.00 |
| rs411093 | 7 | 52007969 | C | T | 10.81 | 1.81 | 2.26E-09 | 0.988 | 1.00 |
| rs411481 | 7 | 52008508 | G | A | 10.81 | 1.81 | 2.26E-09 | 0.988 | 1.00 |
| rs402450 | 7 | 52008755 | A | G | 10.81 | 1.81 | 2.26E-09 | 0.988 | 1.00 |
| rs455017 | 7 | 52002774 | C | T | 10.86 | 1.82 | 2.27E-09 | 0.988 | 0.99 |
| rs396471 | 7 | 52005401 | G | T | 10.84 | 1.81 | 2.28E-09 | 0.988 | 1.00 |
| rs590600 | 7 | 52004473 | A | T | 10.85 | 1.81 | 2.30E-09 | 0.988 | 0.99 |
| rs384551 | 7 | 52001102 | A | G | 10.86 | 1.82 | 2.31E-09 | 0.988 | 0.99 |
| rs409776 | 7 | 52001673 | T | G | 10.86 | 1.82 | 2.31E-09 | 0.988 | 0.99 |
| rs408595 | 7 | 52000754 | C | T | 10.86 | 1.82 | 2.31E-09 | 0.988 | 0.99 |
| rs608826 | 7 | 52000958 | G | A | 10.86 | 1.82 | 2.32E-09 | 0.988 | 0.99 |
| rs438867 | 7 | 51999538 | T | C | 10.87 | 1.82 | 2.32E-09 | 0.988 | 0.99 |
| rs382727 | 7 | 51998920 | C | T | 10.87 | 1.82 | 2.33E-09 | 0.988 | 0.99 |
| rs379235 | 7 | 51998152 | A | T | 10.87 | 1.82 | 2.33E-09 | 0.988 | 0.99 |
| rs426522 | 7 | 51997533 | T | C | 10.87 | 1.82 | 2.34E-09 | 0.988 | 0.99 |
| rs454404 | 7 | 51996191 | C | T | 10.99 | 1.84 | 2.46E-09 | 0.989 | 0.98 |
| rs452777 | 7 | 51995163 | C | T | 10.99 | 1.84 | 2.50E-09 | 0.989 | 0.98 |
| rs10480946 | 7 | 52009321 | C | T | 10.70 | 1.80 | 2.64E-09 | 0.988 | 0.99 |
| rs6971505 | 7 | 52009980 | A | T | 10.90 | 1.86 | 5.14E-09 | 0.989 | 1.00 |
| rs400204 | 7 | 52014571 | A | G | 10.90 | 1.86 | 5.15E-09 | 0.989 | 1.00 |
| rs424283 | 7 | 52023670 | A | G | 10.90 | 1.86 | 5.16E-09 | 0.989 | 1.00 |
| rs201492164 | 7 | 51999316 | T | TTG | 10.44 | 1.81 | 8.08E-09 | 0.988 | 0.96 |
| rs664926 | 7 | 52016205 | A | T | 10.02 | 1.74 | 8.17E-09 | 0.987 | 1.00 |
| rs647653 | 7 | 52042739 | C | T | 9.67 | 1.73 | 2.07E-08 | 0.987 | 0.99 |
| rs428955 | 7 | 52042976 | T | A | 9.54 | 1.73 | 3.24E-08 | 0.987 | 0.97 |
| rs201423711 | 7 | 51999664 | AT | A | 8.77 | 1.59 | 3.52E-08 | 0.981 | 0.81 |
| rs11405039 | 7 | 51999666 | TG | T | 8.77 | 1.59 | 3.52E-08 | 0.981 | 0.81 |

**Table S12. Genomewide significant associations with total questionnaire in mothers**

| **SNP** | **CHR** | **BP** | **A1** | **A2** | **Beta** | **SE** | **P** | **A1_freq** | **info** |
| --- | --- | --- | --- | --- | --- | --- | --- | --- | --- |
| rs10626545 | 7 | 51999752 | TCA | T | 9.65 | 1.57 | 8.55E-10 | 0.987 | 0.94 |
| rs406001 | 7 | 52006415 | C | T | 9.53 | 1.56 | 1.05E-09 | 0.988 | 1.00 |
| rs392252 | 7 | 52039815 | G | A | 9.55 | 1.57 | 1.22E-09 | 0.988 | 1.00 |
| rs10234957 | 7 | 52011171 | A | G | 9.51 | 1.56 | 1.27E-09 | 0.988 | 1.00 |
| rs10265236 | 7 | 52011178 | T | C | 9.51 | 1.56 | 1.27E-09 | 0.988 | 1.00 |
| rs382903 | 7 | 52033199 | A | G | 9.50 | 1.57 | 1.34E-09 | 0.988 | 1.00 |
| rs413648 | 7 | 52013580 | G | T | 9.50 | 1.57 | 1.34E-09 | 0.988 | 1.00 |
| rs377851 | 7 | 52031652 | T | A | 9.50 | 1.57 | 1.35E-09 | 0.988 | 1.00 |
| rs403531 | 7 | 52007845 | G | A | 9.50 | 1.57 | 1.35E-09 | 0.988 | 1.00 |
| rs616199 | 7 | 52014884 | C | A | 9.50 | 1.57 | 1.35E-09 | 0.988 | 1.00 |
| rs428360 | 7 | 52015414 | A | T | 9.50 | 1.57 | 1.35E-09 | 0.988 | 1.00 |
| rs410473 | 7 | 52015969 | A | G | 9.50 | 1.57 | 1.35E-09 | 0.988 | 1.00 |
| rs364752 | 7 | 52021977 | T | C | 9.50 | 1.57 | 1.36E-09 | 0.988 | 1.00 |
| rs417388 | 7 | 52029191 | T | C | 9.50 | 1.57 | 1.36E-09 | 0.988 | 1.00 |
| rs454526 | 7 | 52030332 | C | A | 9.50 | 1.57 | 1.36E-09 | 0.988 | 1.00 |
| rs455017 | 7 | 52002774 | C | T | 9.55 | 1.57 | 1.36E-09 | 0.988 | 0.99 |
| rs411093 | 7 | 52007969 | C | T | 9.50 | 1.57 | 1.36E-09 | 0.988 | 1.00 |
| rs411481 | 7 | 52008508 | G | A | 9.50 | 1.57 | 1.36E-09 | 0.988 | 1.00 |
| rs402450 | 7 | 52008755 | A | G | 9.50 | 1.57 | 1.36E-09 | 0.988 | 1.00 |
| rs396471 | 7 | 52005401 | G | T | 9.53 | 1.57 | 1.37E-09 | 0.988 | 1.00 |
| rs590600 | 7 | 52004473 | A | T | 9.54 | 1.57 | 1.38E-09 | 0.988 | 0.99 |
| rs384551 | 7 | 52001102 | A | G | 9.55 | 1.58 | 1.38E-09 | 0.988 | 0.99 |
| rs409776 | 7 | 52001673 | T | G | 9.55 | 1.57 | 1.38E-09 | 0.988 | 0.99 |
| rs408595 | 7 | 52000754 | C | T | 9.55 | 1.58 | 1.38E-09 | 0.988 | 0.99 |
| rs608826 | 7 | 52000958 | G | A | 9.55 | 1.58 | 1.39E-09 | 0.988 | 0.99 |
| rs10480946 | 7 | 52009321 | C | T | 9.44 | 1.56 | 1.39E-09 | 0.988 | 0.99 |
| rs438867 | 7 | 51999538 | T | C | 9.55 | 1.58 | 1.39E-09 | 0.988 | 0.99 |
| rs382727 | 7 | 51998920 | C | T | 9.55 | 1.58 | 1.39E-09 | 0.988 | 0.99 |
| rs379235 | 7 | 51998152 | A | T | 9.56 | 1.58 | 1.39E-09 | 0.988 | 0.99 |
| rs426522 | 7 | 51997533 | T | C | 9.56 | 1.58 | 1.39E-09 | 0.988 | 0.99 |
| rs454404 | 7 | 51996191 | C | T | 9.67 | 1.60 | 1.43E-09 | 0.989 | 0.98 |
| rs452777 | 7 | 51995163 | C | T | 9.67 | 1.60 | 1.46E-09 | 0.989 | 0.98 |
| rs6971505 | 7 | 52009980 | A | T | 9.71 | 1.62 | 1.99E-09 | 0.989 | 1.00 |
| rs400204 | 7 | 52014571 | A | G | 9.71 | 1.62 | 1.99E-09 | 0.989 | 1.00 |
| rs424283 | 7 | 52023670 | A | G | 9.71 | 1.62 | 2.00E-09 | 0.989 | 1.00 |
| rs201492164 | 7 | 51999316 | T | TTG | 9.18 | 1.57 | 4.96E-09 | 0.988 | 0.96 |
| rs664926 | 7 | 52016205 | A | T | 8.77 | 1.51 | 5.90E-09 | 0.987 | 1.00 |
| rs647653 | 7 | 52042739 | C | T | 8.53 | 1.50 | 1.18E-08 | 0.987 | 0.99 |
| rs428955 | 7 | 52042976 | T | A | 8.42 | 1.50 | 1.82E-08 | 0.987 | 0.97 |
| rs201423711 | 7 | 51999664 | AT | A | 7.76 | 1.38 | 1.87E-08 | 0.981 | 0.81 |
| rs11405039 | 7 | 51999666 | TG | T | 7.76 | 1.38 | 1.87E-08 | 0.981 | 0.81 |

**Table S13. Genomewide significant associations with mother questionnaire in mothers**

| **SNP** | **CHR** | **BP** | **A1** | **A2** | **Beta** | **SE** | **P** | **A1_freq** | **info** |
| --- | --- | --- | --- | --- | --- | --- | --- | --- | --- |
| rs406001 | 7 | 52006415 | C | T | 2.27 | 0.39 | 8.27E-09 | 0.988 | 1.00 |
| rs392252 | 7 | 52039815 | G | A | 2.27 | 0.40 | 9.84E-09 | 0.988 | 1.00 |
| rs10234957 | 7 | 52011171 | A | G | 2.26 | 0.39 | 9.92E-09 | 0.988 | 1.00 |
| rs10265236 | 7 | 52011178 | T | C | 2.26 | 0.39 | 9.92E-09 | 0.988 | 1.00 |
| rs413648 | 7 | 52013580 | G | T | 2.26 | 0.39 | 1.07E-08 | 0.988 | 1.00 |
| rs382903 | 7 | 52033199 | A | G | 2.26 | 0.39 | 1.07E-08 | 0.988 | 1.00 |
| rs403531 | 7 | 52007845 | G | A | 2.26 | 0.39 | 1.07E-08 | 0.988 | 1.00 |
| rs616199 | 7 | 52014884 | C | A | 2.26 | 0.39 | 1.07E-08 | 0.988 | 1.00 |
| rs377851 | 7 | 52031652 | T | A | 2.26 | 0.39 | 1.07E-08 | 0.988 | 1.00 |
| rs428360 | 7 | 52015414 | A | T | 2.26 | 0.39 | 1.07E-08 | 0.988 | 1.00 |
| rs410473 | 7 | 52015969 | A | G | 2.26 | 0.39 | 1.07E-08 | 0.988 | 1.00 |
| rs364752 | 7 | 52021977 | T | C | 2.26 | 0.39 | 1.08E-08 | 0.988 | 1.00 |
| rs417388 | 7 | 52029191 | T | C | 2.26 | 0.39 | 1.08E-08 | 0.988 | 1.00 |
| rs454526 | 7 | 52030332 | C | A | 2.26 | 0.39 | 1.08E-08 | 0.988 | 1.00 |
| rs411093 | 7 | 52007969 | C | T | 2.26 | 0.39 | 1.08E-08 | 0.988 | 1.00 |
| rs411481 | 7 | 52008508 | G | A | 2.26 | 0.39 | 1.08E-08 | 0.988 | 1.00 |
| rs402450 | 7 | 52008755 | A | G | 2.26 | 0.39 | 1.08E-08 | 0.988 | 1.00 |
| rs10480946 | 7 | 52009321 | C | T | 2.25 | 0.39 | 1.09E-08 | 0.988 | 0.99 |
| rs455017 | 7 | 52002774 | C | T | 2.27 | 0.40 | 1.09E-08 | 0.988 | 0.99 |
| rs396471 | 7 | 52005401 | G | T | 2.27 | 0.40 | 1.09E-08 | 0.988 | 1.00 |
| rs590600 | 7 | 52004473 | A | T | 2.27 | 0.40 | 1.10E-08 | 0.988 | 0.99 |
| rs384551 | 7 | 52001102 | A | G | 2.27 | 0.40 | 1.11E-08 | 0.988 | 0.99 |
| rs408595 | 7 | 52000754 | C | T | 2.27 | 0.40 | 1.11E-08 | 0.988 | 0.99 |
| rs409776 | 7 | 52001673 | T | G | 2.27 | 0.40 | 1.11E-08 | 0.988 | 0.99 |
| rs608826 | 7 | 52000958 | G | A | 2.27 | 0.40 | 1.11E-08 | 0.988 | 0.99 |
| rs438867 | 7 | 51999538 | T | C | 2.27 | 0.40 | 1.11E-08 | 0.988 | 0.99 |
| rs382727 | 7 | 51998920 | C | T | 2.27 | 0.40 | 1.12E-08 | 0.988 | 0.99 |
| rs379235 | 7 | 51998152 | A | T | 2.27 | 0.40 | 1.12E-08 | 0.988 | 0.99 |
| rs426522 | 7 | 51997533 | T | C | 2.27 | 0.40 | 1.12E-08 | 0.988 | 0.99 |
| rs10626545 | 7 | 51999752 | TCA | T | 2.26 | 0.40 | 1.12E-08 | 0.987 | 0.94 |
| rs454404 | 7 | 51996191 | C | T | 2.30 | 0.40 | 1.18E-08 | 0.989 | 0.98 |
| rs452777 | 7 | 51995163 | C | T | 2.30 | 0.40 | 1.20E-08 | 0.989 | 0.98 |
| rs6971505 | 7 | 52009980 | A | T | 2.32 | 0.41 | 1.33E-08 | 0.989 | 1.00 |
| rs400204 | 7 | 52014571 | A | G | 2.32 | 0.41 | 1.34E-08 | 0.989 | 1.00 |
| rs424283 | 7 | 52023670 | A | G | 2.32 | 0.41 | 1.34E-08 | 0.989 | 1.00 |
| rs201492164 | 7 | 51999316 | T | TTG | 2.18 | 0.40 | 3.63E-08 | 0.988 | 0.96 |
| rs664926 | 7 | 52016205 | A | T | 2.07 | 0.38 | 4.79E-08 | 0.987 | 1.00 |

**Table S14. Genomewide significant associations with total participation in children**

| **SNP** | **CHR** | **BP** | **A1** | **A2** | **Beta** | **SE** | **P** | **A1_freq** | **info** |
| --- | --- | --- | --- | --- | --- | --- | --- | --- | --- |
| rs28631073 | 14 | 96726466 | G | A | -3.20 | 0.57 | 2.27E-08 | 0.11 | 1.00 |
| rs10143977 | 14 | 96726883 | G | T | -3.19 | 0.57 | 2.35E-08 | 0.11 | 1.00 |
| rs10134118 | 14 | 96726986 | T | C | -3.18 | 0.57 | 2.85E-08 | 0.11 | 1.00 |
| rs11622768 | 14 | 96727554 | T | C | -3.17 | 0.57 | 3.12E-08 | 0.11 | 1.00 |
| rs10137303 | 14 | 96728002 | G | A | -3.15 | 0.57 | 3.52E-08 | 0.11 | 1.00 |
| rs10147171 | 14 | 96727874 | C | T | -3.14 | 0.57 | 4.09E-08 | 0.11 | 1.00 |
| rs4905475 | 14 | 96721850 | C | G | -3.16 | 0.58 | 4.17E-08 | 0.11 | 0.99 |

**Table S15. Genomewide significant associations with total questionnaire in children**

| **SNP** | **CHR** | **BP** | **A1** | **A2** | **Beta** | **SE** | **P** | **A1_freq** | **info** |
| --- | --- | --- | --- | --- | --- | --- | --- | --- | --- |
| rs28631073 | 14 | 96726466 | G | A | -2.83 | 0.50 | 1.53E-08 | 0.11 | 1.00 |
| rs10143977 | 14 | 96726883 | G | T | -2.82 | 0.50 | 1.59E-08 | 0.11 | 1.00 |
| rs10134118 | 14 | 96726986 | T | C | -2.81 | 0.50 | 1.96E-08 | 0.11 | 1.00 |
| rs11622768 | 14 | 96727554 | T | C | -2.80 | 0.50 | 2.14E-08 | 0.11 | 1.00 |
| rs10137303 | 14 | 96728002 | G | A | -2.78 | 0.50 | 2.48E-08 | 0.11 | 1.00 |
| rs4905475 | 14 | 96721850 | C | G | -2.79 | 0.50 | 2.88E-08 | 0.11 | 0.99 |
| rs10147171 | 14 | 96727874 | C | T | -2.77 | 0.50 | 2.93E-08 | 0.11 | 1.00 |
| rs11628515 | 14 | 96728111 | A | T | -2.76 | 0.50 | 3.79E-08 | 0.11 | 1.00 |
| rs45528931 | 14 | 96729885 | A | G | -2.74 | 0.50 | 4.78E-08 | 0.11 | 0.99 |

| **SNP** | **CHR** | **BP** | **A1** | **A2** | **Beta** | **SE** | **P** | **A1_freq** | **info** |
| --- | --- | --- | --- | --- | --- | --- | --- | --- | --- |
| rs28631073 | 14 | 96726466 | G | A | -1.18 | 0.21 | 1.29E-08 | 0.11 | 1.00 |
| rs10143977 | 14 | 96726883 | G | T | -1.18 | 0.21 | 1.33E-08 | 0.11 | 1.00 |
| rs10134118 | 14 | 96726986 | T | C | -1.17 | 0.21 | 1.79E-08 | 0.11 | 1.00 |
| rs11622768 | 14 | 96727554 | T | C | -1.16 | 0.21 | 2.02E-08 | 0.11 | 1.00 |
| rs10137303 | 14 | 96728002 | G | A | -1.16 | 0.21 | 2.13E-08 | 0.11 | 1.00 |
| rs10147171 | 14 | 96727874 | C | T | -1.15 | 0.21 | 2.69E-08 | 0.11 | 1.00 |
| rs4905475 | 14 | 96721850 | C | G | -1.16 | 0.21 | 2.71E-08 | 0.11 | 0.99 |
| rs11628515 | 14 | 96728111 | A | T | -1.14 | 0.21 | 3.71E-08 | 0.11 | 1.00 |

**Table S16. Genomewide significant associations with child questionnaire in children**

**Table S17. Genomewide significant associations with child clinic in children**

| **SNP** | **CHR** | **BP** | **A1** | **A2** | **Beta** | **SE** | **P** | **A1_freq** | **info** |
| --- | --- | --- | --- | --- | --- | --- | --- | --- | --- |
| rs1336852 | 1 | 191752825 | G | A | -0.59 | 0.11 | 3.15E-08 | 0.07 | 0.98 |
| rs74626786 | 1 | 191759598 | G | C | -0.59 | 0.11 | 3.32E-08 | 0.07 | 0.98 |

**Table S17. SNP-based heritability estimates from GCTA**

|  | **H^2^** | **Standard error** | **P-value** |
| --- | --- | --- | --- |
| **Mother** |  |  |  |
| Total participation | 0.271 | 0.045 | 7.00E-11 |
| Total questionnaire | 0.267 | 0.045 | 1.53E-19 |
| Mother questionnaire | 0.238 | 0.044 | 8.59E-09 |
| Mother last clinic | 0.225 | 0.045 | 9.70E-08 |
| Mother last questionnaire | 0.203 | 0.045 | 1.46E-06 |
|  |  |  |  |
| **Child** |  |  |  |
| Total participation | 0.301 | 0.045 | 6.05E-13 |
| Total questionnaire | 0.287 | 0.045 | 7.03E-12 |
| Child questionnaire | 0.319 | 0.045 | 6.06E-14 |
| Child clinic | 0.249 | 0.044 | 9.10E-10 |
| Child last clinic | 0.249 | 0.045 | 4.00E-09 |
| Child last questionnaire | 0.178 | 0.045 | 2.52E-05 |

Adjusted for the first 10 genetic principal components.

**Figure S1. Flowchart of ALSPAC mothers included in analysis**

**
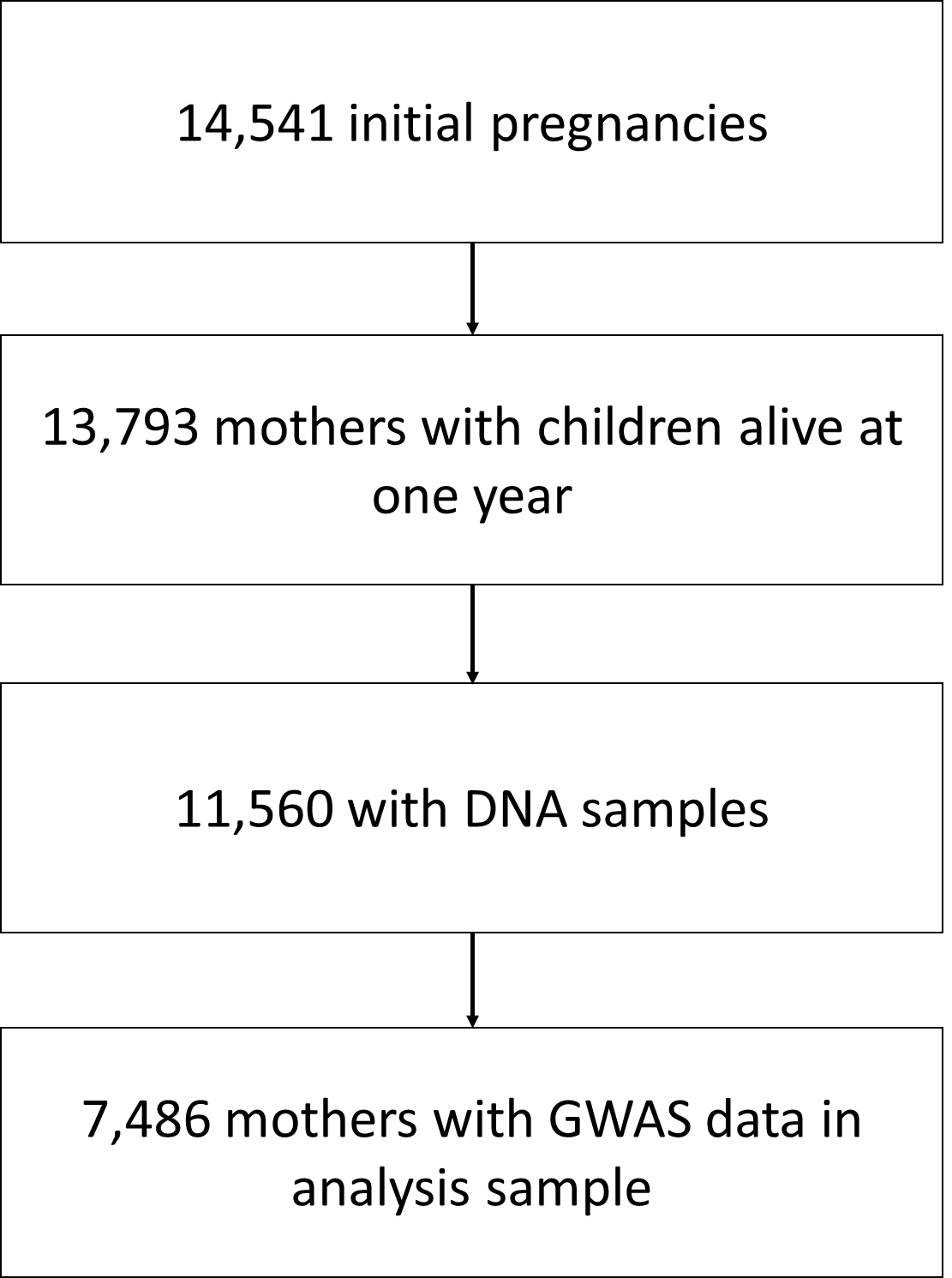
**

**Figure S2. Flowchart of ALSPAC children included in analysis**


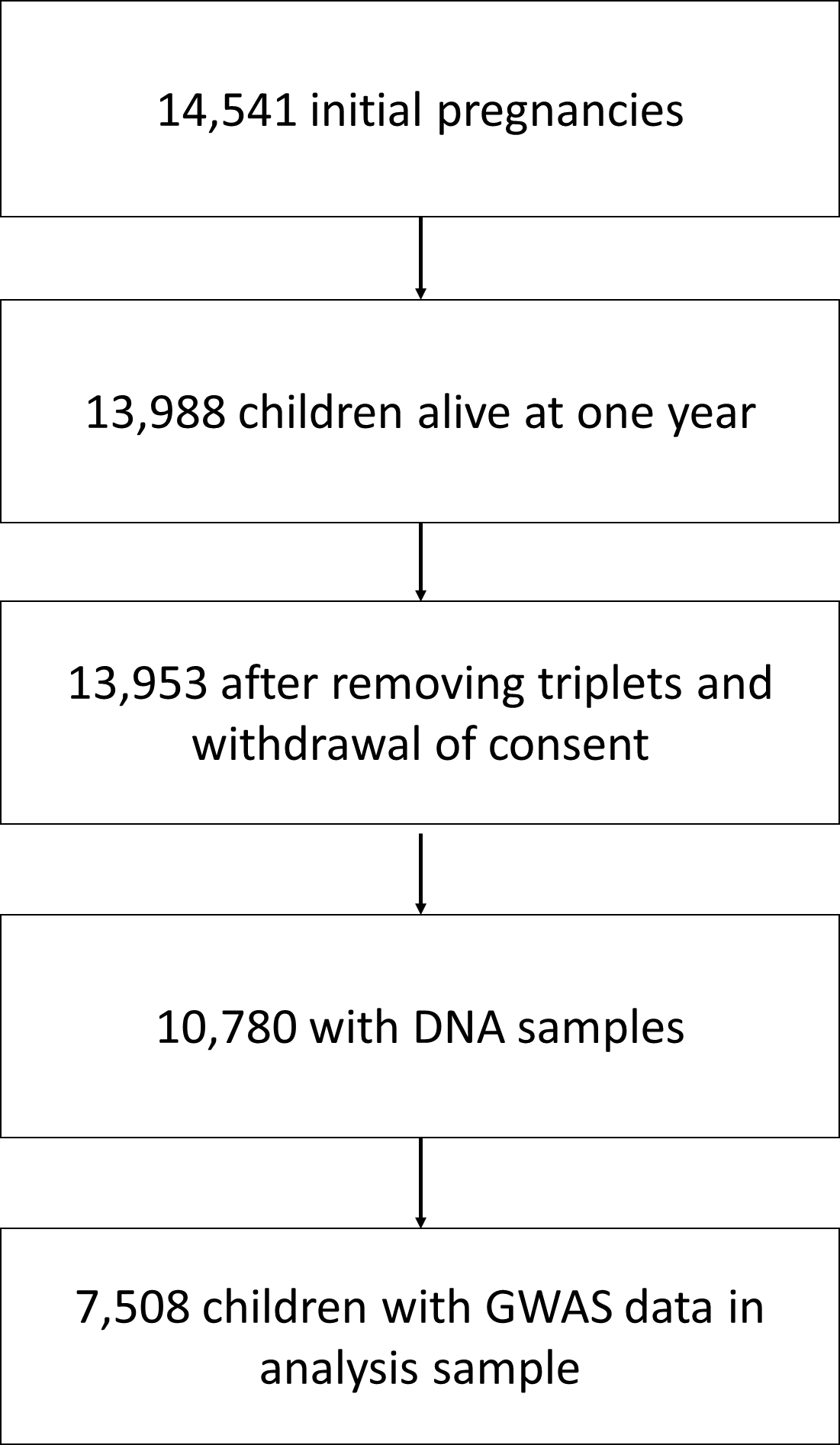


**Figure S3. Associations between polygenic risk scores and attendance at most recent clinic in ALSPAC mothers (N=7,468)**

**
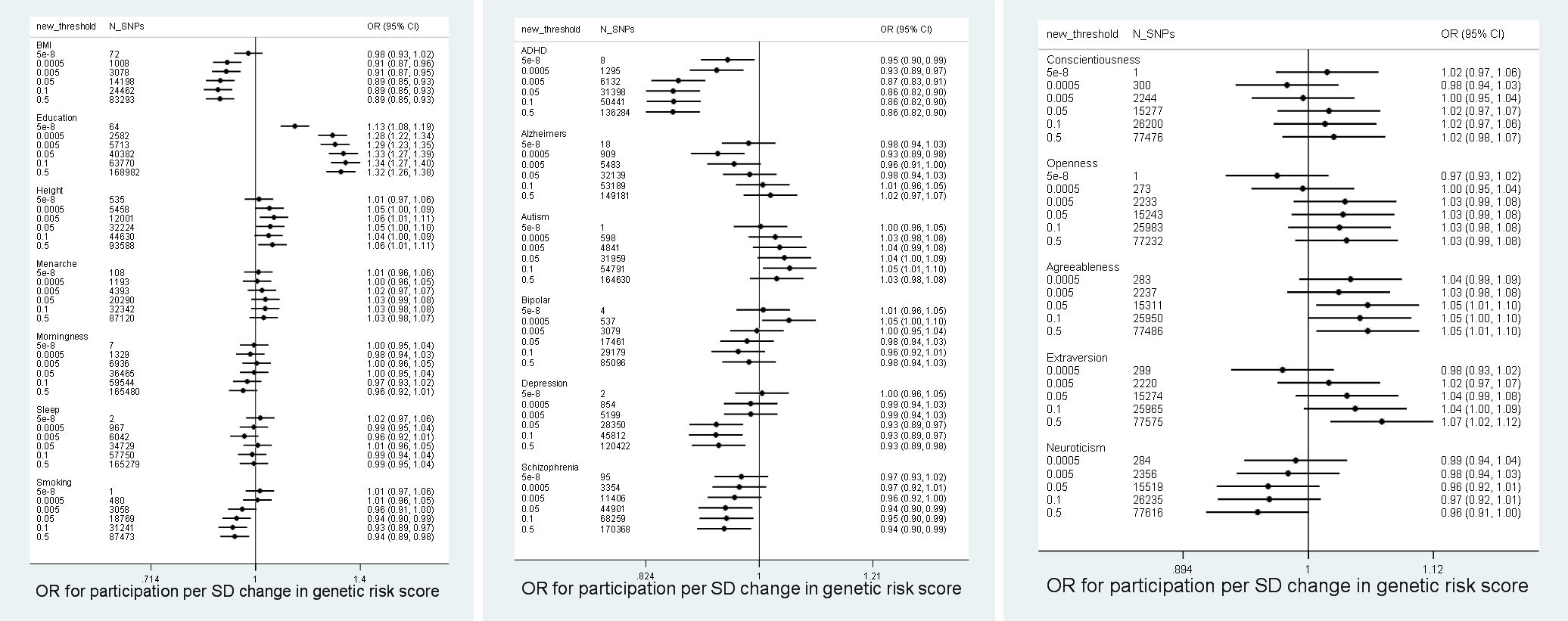
**

**Figure S4. Associations between polygenic risk scores and total questionnaire completion in the mothers (N=7,468)**

**
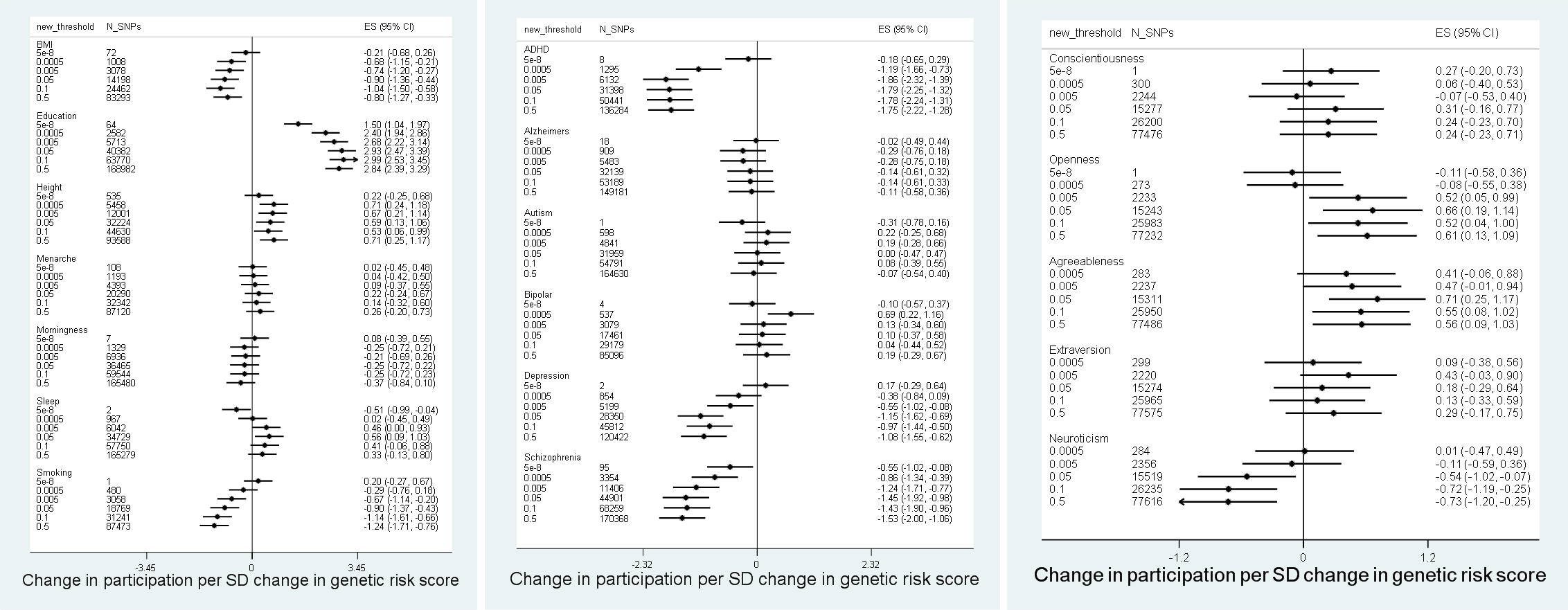
**

**Figure S5. Associations between polygenic risk scores and mother questionnaires (N=7,468)**

**
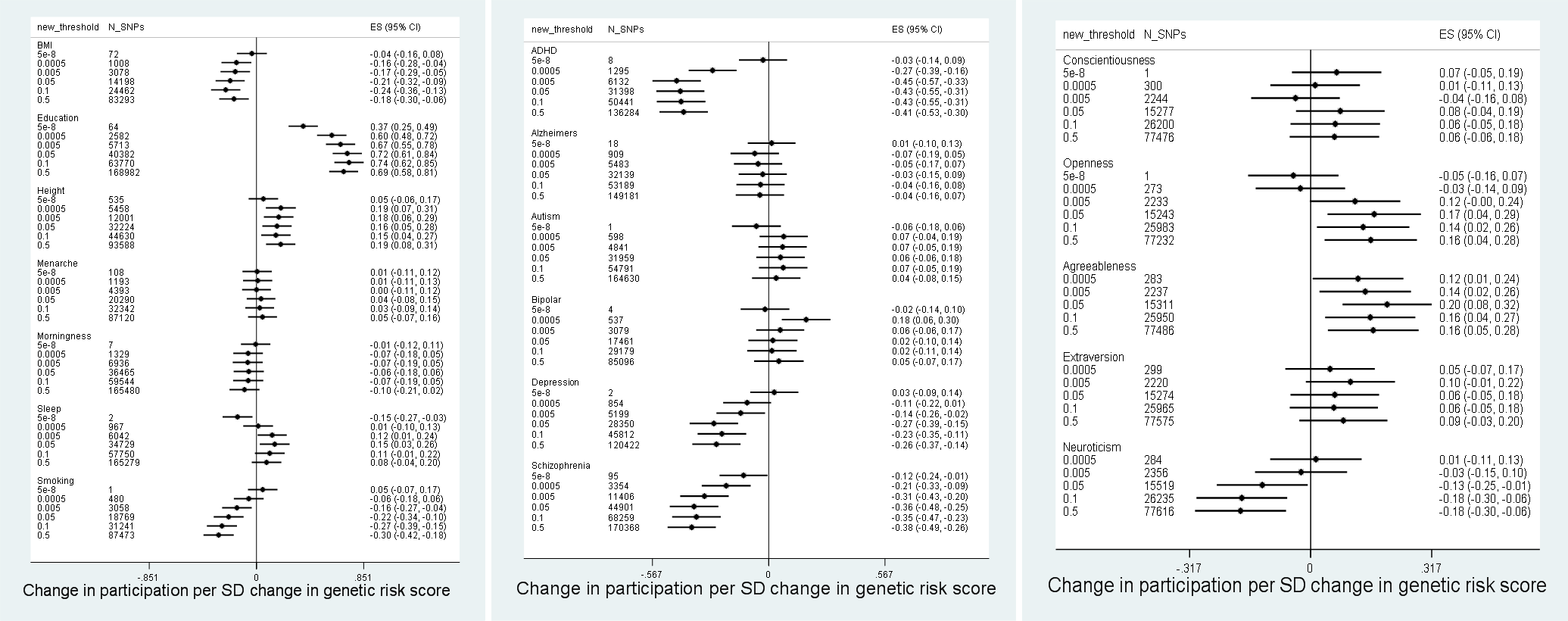
**

**Figure S6. Associations between polygenic risk scores and attendance at most recent clinic in ALSPAC children (N=7,508)**

**
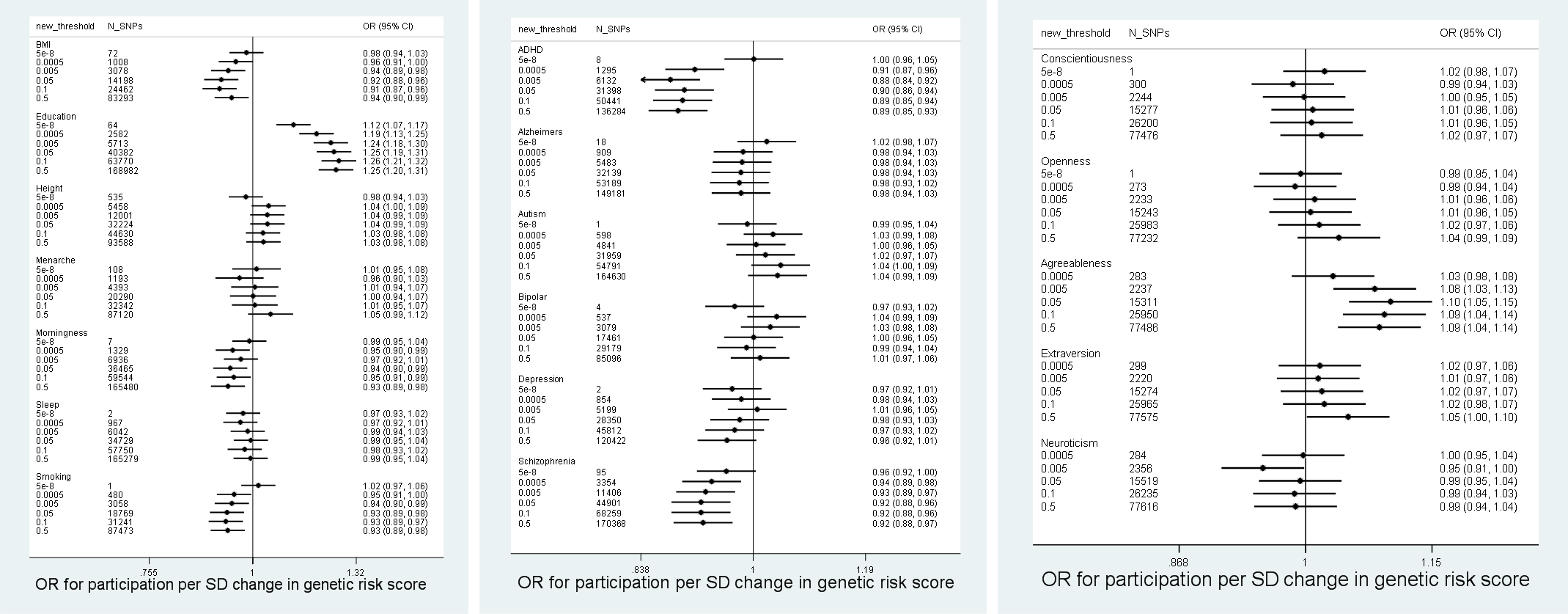
**

Age at menarche analysis only in females

**Figure S7. Associations between polygenic risk scores and total questionnaire completion in ALSPAC children (N=7,508)**

**
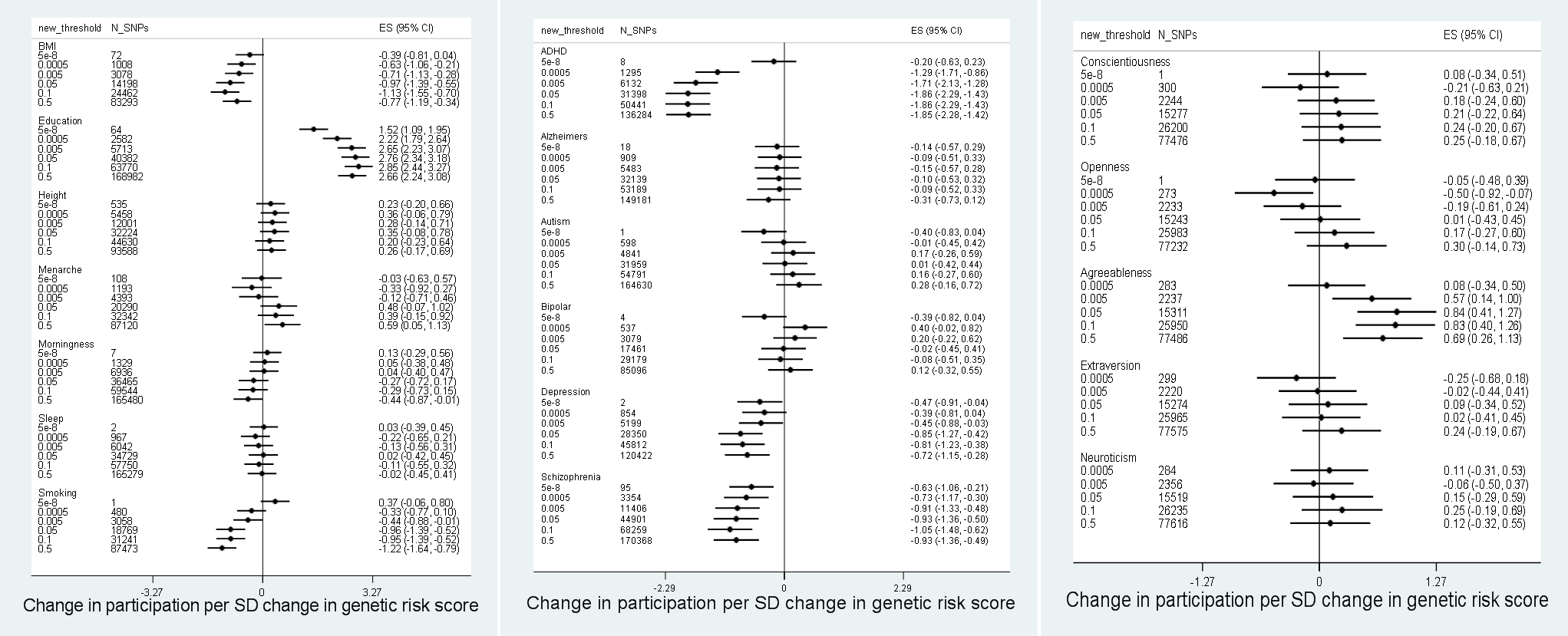
**

Age at menarche analysis only in females

**Figure S8. . Associations between polygenic risk scores and child completed questionnnaires in ALSPAC children (N=7,508)**

**
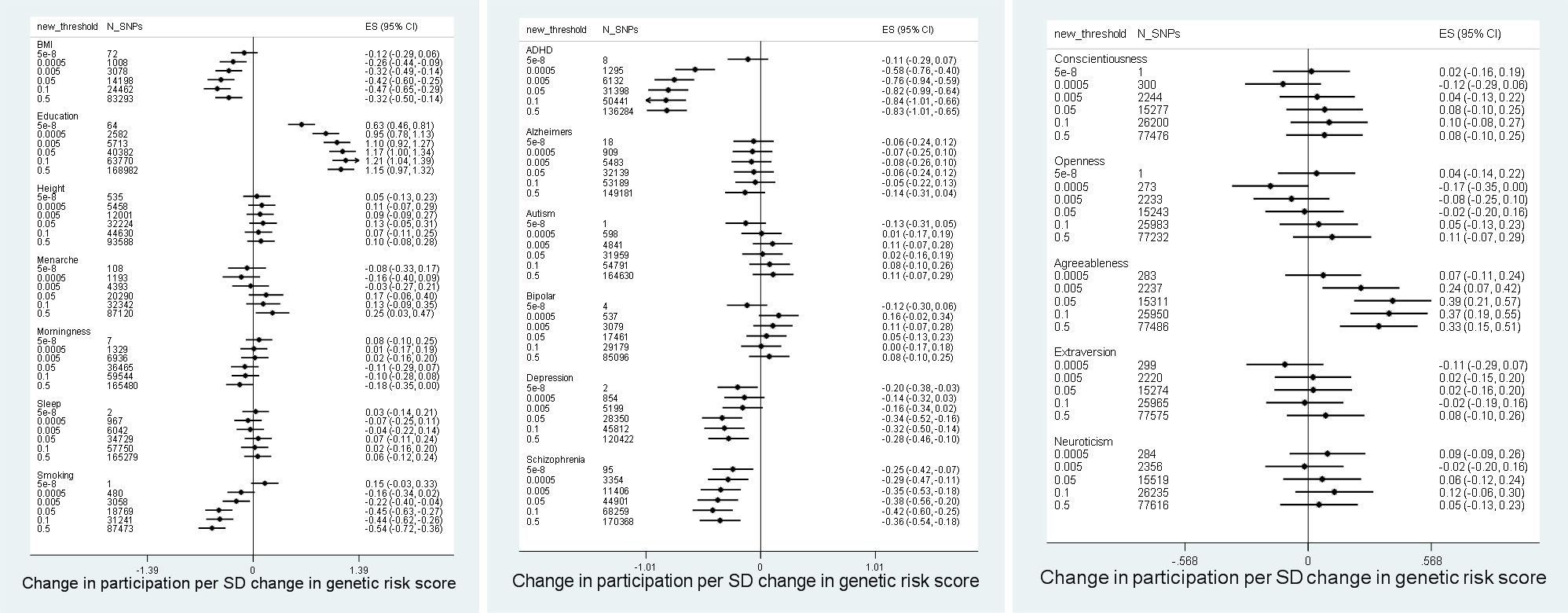
**

Age at menarche analysis only in females

**Figure S9. . Associations between polygenic risk scores and total number of clinics attended in ALSPAC children (N=7,508)**

**
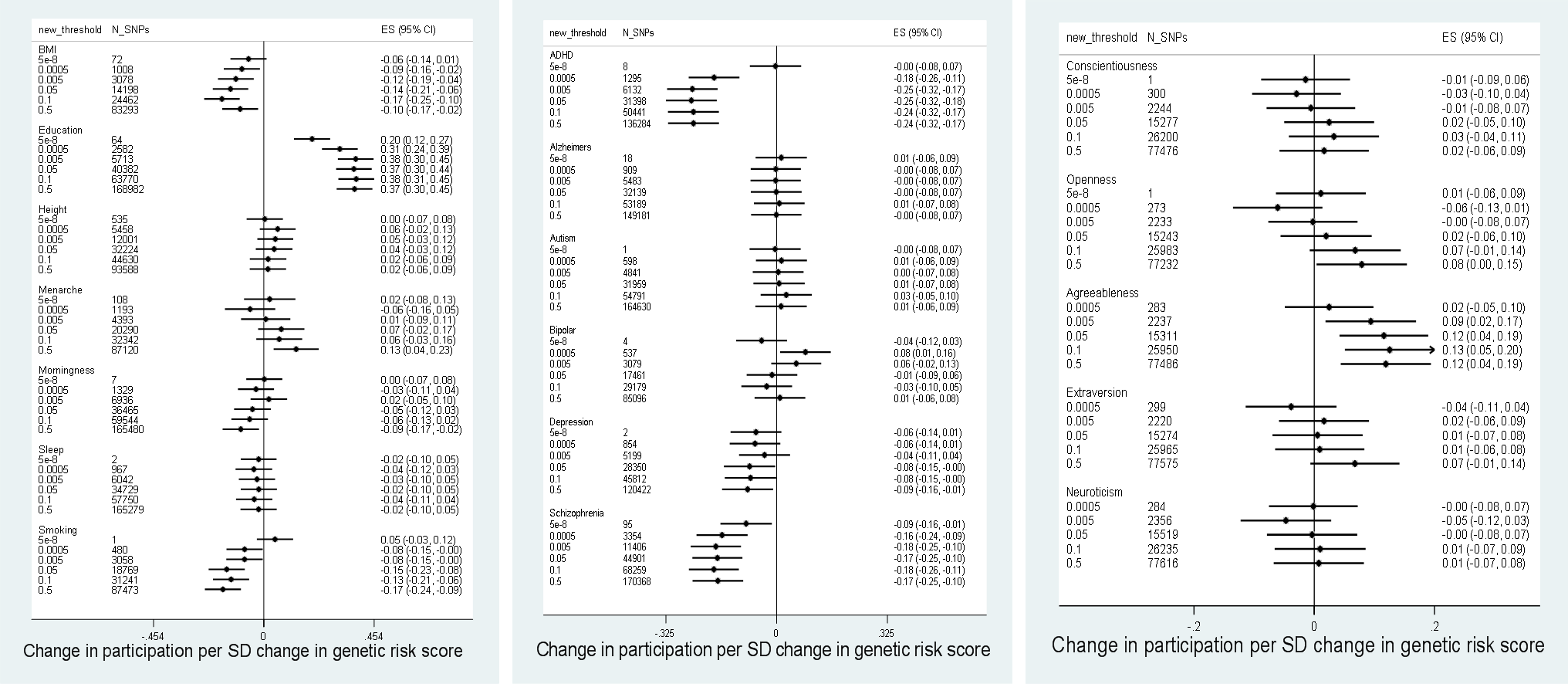
**

**Figure S10. Manhattan plots for GWAS of mother last clinic and mother last questionnaire**

**
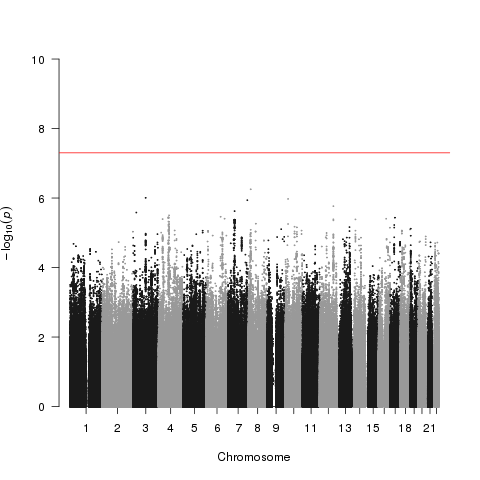

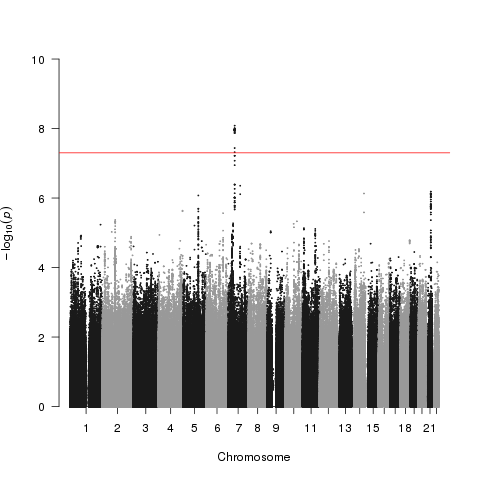

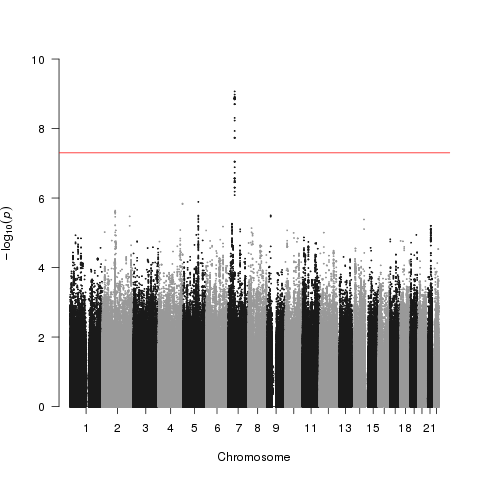

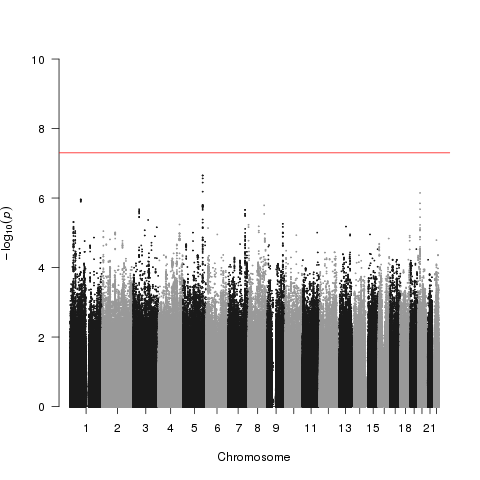
**

A

B

C

D

B

A

1. **Mother total questionnaire, B) Mother questionnaire, C) Mother last clinic, D) Mother last questionnaire**

**Figure S11. QQ plots for GWAS of total participation, total questionnaire and mother questionnaire in the ALSPAC mothers**

**
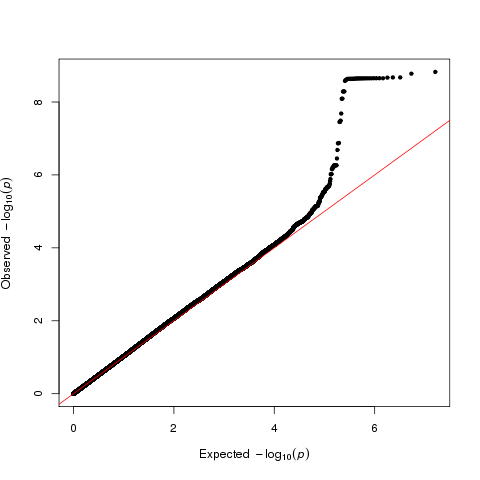

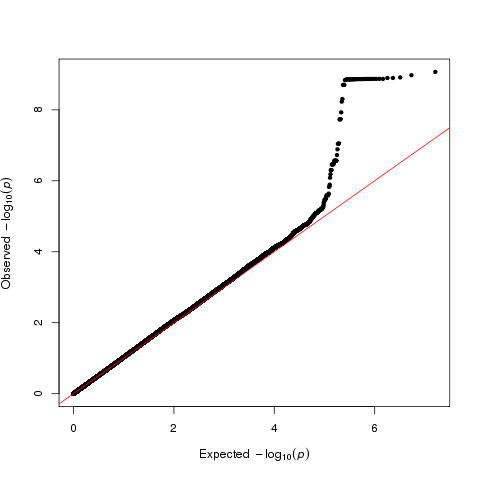

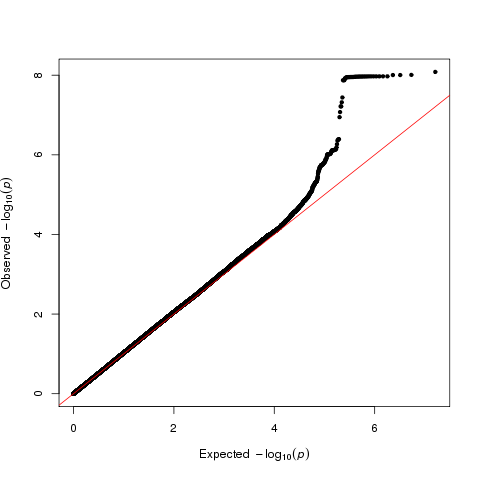
**

C

B

A

1. **Total participation, B) Total questionnaire, C) Mother questionnaire**

**
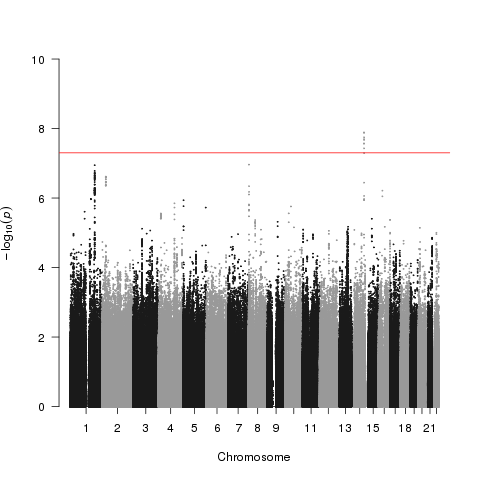

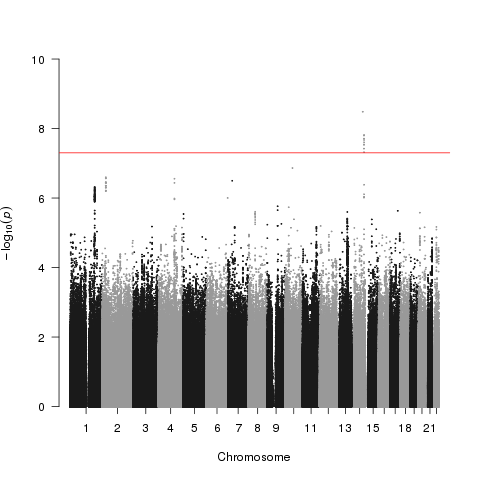

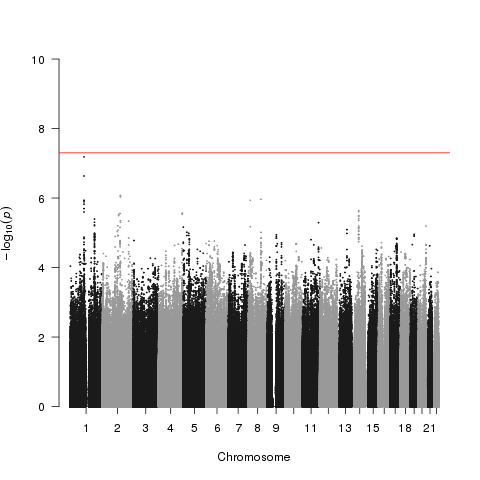

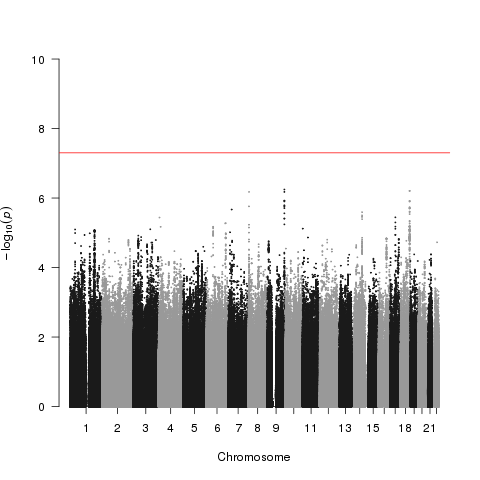
Figure S12. Manhattan plots for GWAS of participation in children**

A

**B**

D

C

1. **Total questionnaire, B) Child questionnaire, C) Child last questionnaire, D) Child last clinic**

**
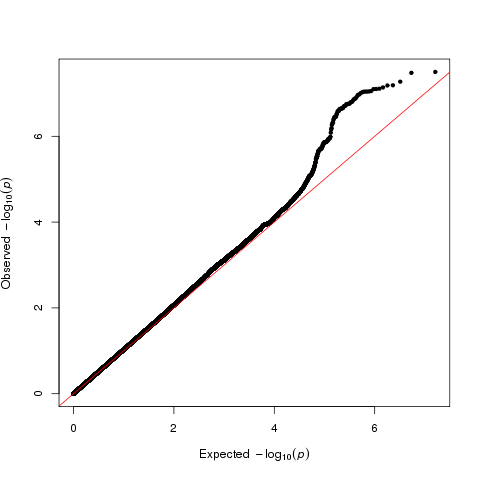

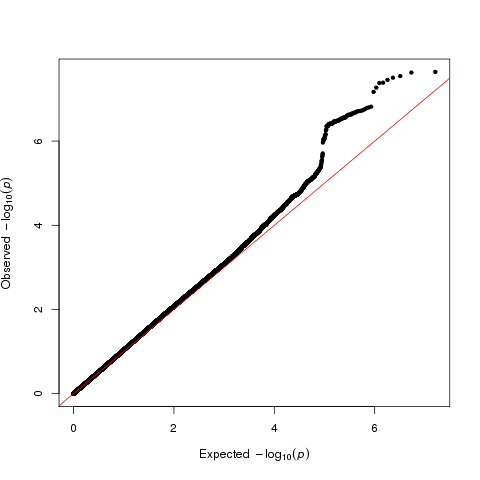
Figure S13. QQ plots for GWAS of total participation, total questionnaire and mother questionnaire in the ALSPAC mothers**

D

C

**B**

A


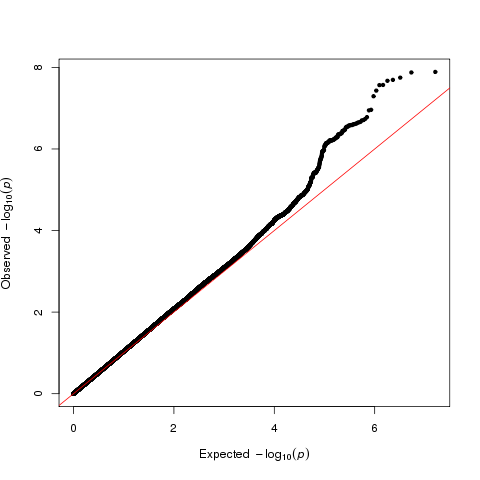
**
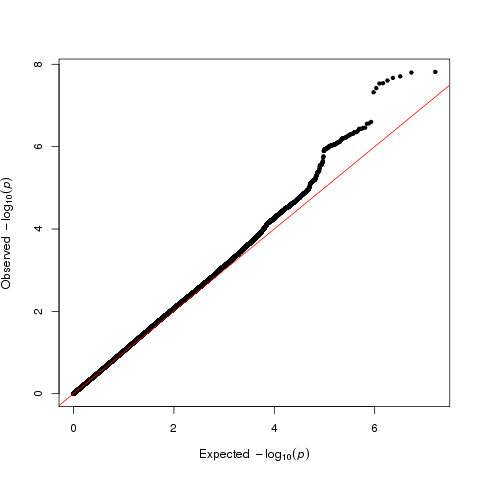
**

1. **Total participation, B) Total questionnaire C) Child questionnaire, D) Child clinic**

**Figure S14. Regional plot of genomewide significant locus for mother total participation**

The SNP with the second smallest p-value was used as LD information was not available for the top SNP. The top SNP in each region is highlighted in purple and the surrounding SNPs are colour coded to reflect their LD with this variant. Estimated recombination rates are plotted in blue to reflect local LD structure. Regional plots generated using Locus Zoom, Genome build= hg19.

**Figure S15. Regional plot of genomewide significant locus for mother total questionnaire**

The SNP with the second smallest p-value was used as LD information was not available for the top SNP. The top SNP in each region is highlighted in purple and the surrounding SNPs are colour coded to reflect their LD with this variant. Estimated recombination rates are plotted in blue to reflect local LD structure. Regional plots generated using Locus Zoom, Genome build= hg19.

**Figure S16. Regional plot of genomewide significant locus for mother questionnaire**

The top SNP in each region is highlighted in purple and the surrounding SNPs are colour coded to reflect their LD with this variant. Estimated recombination rates are plotted in pale blue to reflect local LD structure. Regional plots generated using Locus Zoom, Genome build= hg19.

**Figure S17. Regional plot of genomewide significant locus for child clinic attendance**

The top SNP in each region is highlighted in purple and the surrounding SNPs are colour coded to reflect their LD with this variant. Estimated recombination rates are plotted in pale blue to reflect local LD structure. Regional plots generated using Locus Zoom, Genome build= hg19.

**Figure S18. Regional plot of genomewide significant locus for child total participation**

The top SNP in each region is highlighted in purple and the surrounding SNPs are colour coded to reflect their LD with this variant. Estimated recombination rates are plotted in pale blue to reflect local LD structure. Regional plots generated using Locus Zoom, Genome build= hg19.

**Figure S19. Regional plot of genomewide significant locus for child total questionnaire**

The top SNP in each region is highlighted in purple and the surrounding SNPs are colour coded to reflect their LD with this variant. Estimated recombination rates are plotted in pale blue to reflect local LD structure. Regional plots generated using Locus Zoom, Genome build= hg19.

**Figure S20. Regional plot of genomewide significant locus for child questionnaire**

**References**

1. A. E. Locke *et al.*, Genetic studies of body mass index yield new insights for obesity biology. *Nature* **518**, 197-206 (2015).

2. A. R. Wood *et al.*, Defining the role of common variation in the genomic and biological architecture of adult human height. *Nature genetics* **46**, 1173-1186 (2014).

3. H. Furberg *et al.*, Genome-wide meta-analyses identify multiple loci associated with smoking behavior. *Nature genetics* **42**, 441-U134 (2010).

4. A. Okbay *et al.*, Genome-wide association study identifies 74 loci associated with educational attainment. *Nature* **533**, 539-542 (2016).

5. S. E. Jones *et al.*, Genome-Wide Association Analyses in 128,266 Individuals Identifies New Morningness and Sleep Duration Loci. *PLoS genetics* **12**, e1006125 (2016).

6. J. R. Perry *et al.*, Parent-of-origin-specific allelic associations among 106 genomic loci for age at menarche. *Nature* **514**, 92-97 (2014).

7. A. Okbay *et al.*, Genetic variants associated with subjective well-being, depressive symptoms, and neuroticism identified through genome-wide analyses. *Nature genetics* **48**, 624-633 (2016).

8. S. W. G. o. t. P. G. Consortium, Biological insights from 108 schizophrenia-associated genetic loci. *Nature* **511**, 421-427 (2014).

9. D. Demontis *et al.*, Discovery of the first genome-wide significant risk loci for ADHD. *BioRxiv*, (2017).

10. C. Cross-Disorder Group of the Psychiatric Genomics, Identification of risk loci with shared effects on five major psychiatric disorders: a genome-wide analysis. *Lancet* **381**, 1371-1379 (2013).

11. M. H. de Moor *et al.*, Meta-analysis of genome-wide association studies for personality. *Molecular psychiatry* **17**, 337-349 (2012).

12. J. C. Lambert *et al.*, Meta-analysis of 74,046 individuals identifies 11 new susceptibility loci for Alzheimer's disease. *Nature genetics* **45**, 1452-1458 (2013).
